# Adaptation Design Tool for Climate-Smart Management of Coral Reefs and Other Natural Resources

**DOI:** 10.1007/s00267-018-1065-y

**Published:** 2018-06-22

**Authors:** Jordan M. West, Catherine A. Courtney, Anna T. Hamilton, Britt A. Parker, David A. Gibbs, Patricia Bradley, Susan H. Julius

**Affiliations:** 1 Office of Research and Development, U.S. Environmental Protection Agency, 1200 Pennsylvania Ave, NW (8623R), Washington, DC 20460 USA; 2 Tetra Tech, Inc., 737 Bishop St., Suite 2340, Honolulu, HI 96813 USA; 3 Center for Ecological Sciences, Tetra Tech, Inc., 502 W. Cordova Road, Suite C, Santa Fe, NM 87505 USA; 4 Cooperative Institute for Research In Environmental Sciences, University of Colorado Boulder, 325 Broadway R/PSD, DSRC/GD111, Boulder, Colorado, 80305 USA; 5 Oak Ridge Institute for Science and Education (ORISE) Fellow at the U.S. Environmental Protection Agency, 1200 Pennsylvania Ave, NW (8623R), Washington, DC 20460 USA; 6 Center for Ecological Sciences, Tetra Tech, Inc., Owings Mills, MD 21117 USA

**Keywords:** Climate change, Vulnerability, Adaptation planning, Natural resource management, Decision making, Coral reefs

## Abstract

Scientists and managers of natural resources have recognized an urgent need for improved methods and tools to enable effective adaptation of management measures in the face of climate change. This paper presents an Adaptation Design Tool that uses a structured approach to break down an otherwise overwhelming and complex process into tractable steps. The tool contains worksheets that guide users through a series of design considerations for adapting their planned management actions to be more climate-smart given changing environmental stressors. Also provided with other worksheets is a framework for brainstorming new adaptation options in response to climate threats not yet addressed in the current plan. Developed and tested in collaboration with practitioners in Hawai’i and Puerto Rico using coral reefs as a pilot ecosystem, the tool and associated reference materials consist of worksheets, instructions and lessons-learned from real-world examples. On the basis of stakeholder feedback from expert consultations during tool development, we present insights and recommendations regarding how to maximize tool efficiency, gain the greatest value from the thought process, and deal with issues of scale and uncertainty. We conclude by reflecting on how the tool advances the theory and practice of assessment and decision-making science, informs higher level strategic planning, and serves as a platform for a systematic, transparent and inclusive process to tackle the practical implications of climate change for management of natural resources.

## Introduction

As the diverse effects of climate change have become increasingly apparent, natural resource management has grown to include adaptation to current and future climate change stressors in addition to traditional management of local stressors. One community of practice that recognized early on the need to adapt to climate change was coral reef managers. The extent and severity of increasingly frequent heat-induced mortality episodes at the global scale (Done et al. 2003; Hoegh-Guldberg 1999; Hughes et al. 2018; Hughes et al. 2017; Wilkinson and Souter 2008; Wolanski et al. 2017) catalyzed efforts by coral reef researchers and managers to evaluate actions to address the impacts of climate change on reefs. Early recommendations focused on accelerating the implementation of existing management plans to reduce local pressures that impair the ability of corals to withstand thermal stress, and developing coral bleaching assessment information for early detection and monitoring (Aeby et al. 2008; Hendee et al. 2001; Marshall and Schuttenberg 2006; Westmacott et al. 2000). Over time, the urgent need to both adapt existing management actions and adopt new strategies became evident, not only for coral reefs (Anthony et al. 2015; Bellwood et al. 2004; Brown et al. 2013; Edgar et al. 2016; Hughes et al. 2010; Integovernmental Panel on Climate Change (IPCC) 2014; Mumby and Steneck 2008; Sale 2008; U.S. Environmental Protection Agency (EPA) 2007), but also for other systems such as wetlands, streams and rivers, watersheds, and estuaries (Barbour et al. 2004; Chan et al. 2016; Hale et al. 2009; Tecle et al. 2003; West et al. 2009). On the basis of a growing mechanistic understanding of both the direct effects of climate change and the interactions of climate change with other stressors, scientists and practitioners are striving to develop new and adapted management techniques that protect and enhance the ecological properties that underlie resilience (Allen et al. 2011; Cote and Darling 2010; Eason et al. 2016; Fujita et al. 2013; Sasaki et al. 2015; Stein et al. 2014).

However, designing and implementing ‘climate-smart’ management adaptations has proven problematic for a variety of reasons. There has been increasing recognition that top-down regulatory and technology-driven responses are not sufficient to address environmental challenges that occur at multiple spatial scales, unfold over long temporal scales and have possible global implications (Grossarth and Hecht 2007). Furthermore, these challenges may be difficult to define, unstable, and socially complex, have no clear or single solution and extend beyond the understanding of one discipline or the responsibility of one organization (Bradley and Yee 2015; Churchman 1967; National Research Council (NRC) 2012; Rittel and Webber 1973; Stahl 2014). To tackle such ‘wicked’ problems (Rittel and Webber 1973), a variety of conceptual models have been used to help understand the interactions and cause-effect relationships in complex social-ecological systems in order to develop management strategies and actions (Binder et al. 2013; Bradley and Yee 2015; Conservation Measures Partnership 2013). With the continuously growing understanding of climate change risks to coral reefs and other ecosystems, there is a need to revisit these conceptual models through a climate lens and reevaluate the appropriateness or feasibility of existing and planned management activities.

It is in this context that several critical needs were identified by the authors when working with U.S. and international practitioners in natural resource management. In particular, how do we apply climate-smart principles within our current understanding of ecosystem management to redesign and re-engineer management plans and actions to address the new realities posed by climate change? Initially, it was difficult for science and management to move beyond existing conceptual frameworks that have guided management under relatively stable climate conditions for many decades (West et al. 2009). As a result, management remained focused on non-climate stressors and continued to emphasize accelerated implementation. Another limitation was difficulty matching available climate change vulnerability information with management actions. Vulnerability assessments are often produced at a scope and scale (e.g., regional or national) and by individuals or groups not directly linked with natural resource management (which is generally more localized). Thus, vulnerability information has to be summarized and applied from larger scale studies that might not provide appropriate or sufficient information to inform local management. Climate change is associated with many uncertainties (e.g., climate change projections, ecosystem responses) and surprises (e.g. unanticipated threshold changes, unexpected differences in sensitivity to bleaching among coral species). Therefore, managers need a systematic method to account for unpredictable outcomes and support an adaptive management process where continual refinement and adoption of new strategies should become the norm.

Given the continuing crisis surrounding coral reef degradation and associated management needs at multiple scales, our initial focus for adaptation methods has been coral reef systems. At the local scale, coral reefs are threatened by a variety of anthropogenic stressors, including polluted runoff, land-use practices in adjacent watersheds, coastal development and near-shore dredging (Acevedo et al. 1989; Bak 1978; Bejarano and Appeldoorn 2013; Bradley et al. 2009; Center for Watershed Protection (CWP) 2008; Dodge et al. 1974; Dodge and Vaisnys 1977; Fabricius 2005; Hubbard 1986; Rogers 1990; Storlazzi et al. 2015; Vega Thurber et al. 2014; Warne et al. 2005; Wooldridge and Done 2009). At the same time, over-fishing has continued to dramatically alter fish community composition on coral reefs (Appeldoorn and Meyers 1993; Ault et al. 1998; Ault et al. 2008; Brandt et al. 2009; Hay 1984; Hay 1991; Hughes 1994; Jackson 1997; Jackson et al. 2014; Jackson and Sala 2001; Knowlton and Jackson 2008; Mora 2008; Pandolfi et al. 2003; Sala et al. 2001).

At the global scale, consequences for coral reefs of rising atmospheric CO_2_ concentrations include accelerating changes in sea surface temperatures, precipitation patterns, sea level rise and carbonate saturation equilibrium (pH) (Durack and Wijffels 2010; Durack et al. 2012; Hansen et al. 1988; Hansen et al. 2005; Hansen et al. 2000; Hoegh-Guldberg 2014; Integovernmental Panel on Climate Change (IPCC) 2013; Rayner et al. 2003; Rogers and Miller 2006). There is also considerable evidence for increasing intensity of tropical storms in some regions since the 1970s (Integovernmental Panel on Climate Change (IPCC) 2014), which is increasing the force of wave action in coastal areas (Hamylton et al. 2013; Saunders et al. 2016). Due to the combination of these interacting local and global factors, coral reef ecosystems are degrading rapidly (Hoegh-Guldberg 2014; Hoegh-Guldberg et al. 2017; Hughes et al. 2003; Jackson et al. 2014; Jackson et al. 2001; McField and Kramer 2007; Pandolfi et al. 2003; Waddell 2005; Wilkinson 2008; Wilkinson 2004), with declines of 50% or more over the past 30–50 years in large parts of the world’s tropical regions (Bruno and Selig 2007; De’ath et al. 2012; Gardner et al. 2003; Hughes 1994). As mass coral bleaching and mortality events are now occurring globally, there is strong evidence that coral dominated ecosystems will be unable to cope and will continue to disappear without effective management interventions (Done et al. 2003; Eakin et al. 2010; Hoegh-Guldberg 1999).

The Adaptation Design Tool described in this paper was undertaken with the above challenges in mind. Availability of a specifically-applicable tool to help address climate change adaptation will enable natural resource managers to take a systematic approach and engage in more focused evaluation of interactive climate and non-climate impacts on targeted ecosystems and management actions. The Adaptation Design Tool is a product of the Corals & Climate Adaptation Planning (CCAP) project, a collaborative effort of the Climate Change Working Group of the interagency U.S. Coral Reef Task Force, whose mission is to tailor and test general principles of climate-smart adaptation (Stein et al. 2014), specifically for coral reef management. In the first phase of the CCAP project, West et al. (2016) developed a ‘Compendium’ of adaptation options from the literature, to help coral reef managers consider new climate-smart adaptation strategies and management actions as part of their existing management portfolios.

In this paper, we describe the next phase of the project: the development and testing of the Adaptation Design Tool (hereafter called the Design Tool). The Design Tool consists of a structured process that is supported by worksheets (Tables 2, 3 and 4), instructions and examples to help managers (1) analyze and adapt existing management actions in the context of climate-smart design, and (2) identify new adaptation options using the Compendium. The Design Tool was developed and tested over a three-year period in collaboration with coral reef managers, scientists, and practitioners. We begin by providing an overview of the Design Tool in the context of the climate-smart cycle (West et al. 2016), followed by a description of the basic components and workflow for using the tool (Parker et al. 2017)^1^. The results from testing the Design Tool in two coral reef settings--Guánica Bay, Puerto Rico and West Maui, Hawai’i--are compared in order to examine similarities, differences, and lessons learned. Insights gained from this process are discussed in terms of tool effectiveness and efficiency, value-added benefits from the thought process, and overarching issues of scale and uncertainty. Conclusions highlight the tool’s role in advancing assessment theory and practice, informing higher-level strategic planning, and facilitating systematic, transparent and inclusive dialogue to address climate change vulnerabilities.

## Adaptation Design Tool: Overview

### Conceptual Background from Previous Work

**Fig. 1:**
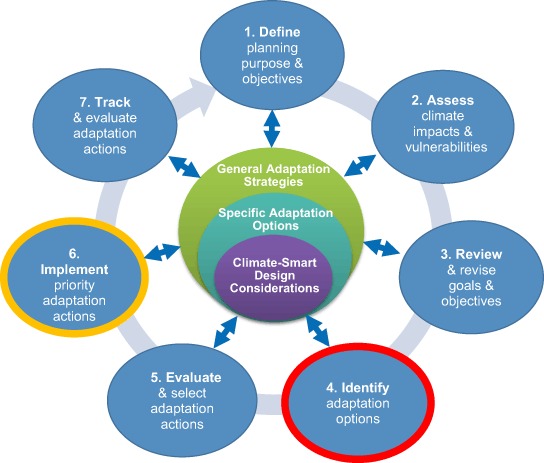
Climate-Smart Cycle with Adaptation Design Framework (Stein et al. 2014; West et al. 2016)

As mentioned, the first phase of the CCAP project (West et al. 2016) adapted the general climate-smart cycle (Stein et al. 2014) and assessed its applicability to coral reefs. Figure 1 shows the adapted climate-smart cycle, which includes general steps in a typical planning process where climate change information can be used to improve management effectiveness. This facilitates accounting for climate change effects in everything from defining goals and objectives; to assessing vulnerabilities from interacting climate and non-climate stressors; to identifying, selecting and implementing climate-smart management responses; to monitoring effectiveness. Making use of extensive vulnerability and resilience information already available for coral reefs (Step 2), we focused on how such information can be applied for brainstorming and designing potential management adaptation options (Step 4) and—once priority actions have been selected (Step 5)—how to further specify more detailed design for implementation (Step 6). While this paper will primarily focus on Step 4, we will touch on Step 6 as well as implications of results for other steps where appropriate. This applicability to multiple steps is consistent with the iterative nature of management planning, where adjustments may become necessary at any step—leading to potential changes in other steps in response--as new and improved information becomes available. These concepts are explored further below.

The adaptation design framework (center of Fig. 1) was used to build a Compendium of adaptation ideas for coral reef managers, drawn from an extensive review of the literature (West et al. 2016). The Compendium organizes information according to seven general adaptation strategies (Box 1) (West and Julius 2014), placing ideas for specific adaptation options into these ‘bins’ to ensure that all categories of approaches are considered. Central to the entire framework is the concept of climate-smart design considerations (Fig. 1). In order to make a generic adaptation idea truly climate-smart for a specific reef system in a particular location, it is necessary to apply two categories of design considerations (Box 2).

Feedback from participants engaged in the initial testing of these general concepts (reported in West et al. (2016)) affirmed the value of addressing climate-smart design considerations for making their watershed and coastal management activities more climate-smart. Participants also recognized the complexity of the considerations raised and articulated a need for an expanded, step-by-step process to break the design consideration concepts (presented in general form in the Compendium) into more detailed and specific place-based questions for their own locations.

The new Design Tool presented in this paper was developed in response to this need. It is structured to aid in ‘unpacking’ climate-smart design considerations into a series of questions that lead the user through the process of brainstorming and crafting climate-smart adaptation actions. As a result, the Design Tool provides a link between climate change vulnerability assessment and the design and implementation of on-the-ground management adaptations that are climate-smart. It is based on the premise that management actions must work in the context of and adjust for global stressors. In Step 4 of the planning cycle (Fig. 1), the Design Tool provides a systematic, transparent and defensible approach to help managers to do the following: (1) integrate climate-smart design considerations into their existing management actions; and (2) build out an expanded set of actions based on options found under general adaptation strategies using information from the literature or from expert consultation.

### Box 1 CCAP Compendium of General Adaptation Strategies, Adaptation Options, and Climate-Smart Design Considerations

The CCAP Compendium (West et al. 2016) provides examples of management adaptation options for coral reef ecosystems compiled from the literature. These are organized using a structure (adapted from West and Julius 2014) for brainstorming and ‘binning’ adaptation options according to categories of ecologically-oriented general strategies familiar to managers, but specifically viewed through the climate change lens. These include:

**Table:** 

**General Strategy**	**Definition**
A. Reduce Non-Climate Stresses	Minimize localized human stressors that hinder the ability of species or ecosystems to withstand or adjust to climatic events
B. Ensure Connectivity	Protect habitats that facilitate movement of organisms (and gene flow) among resource patches
C. Support Evolutionary Potential	Protect a variety of species, populations and ecosystems in multiple places to bet-hedge against losses from climate disturbances, and where possible manage these systems to assist positive evolutionary change
D. Protect Key Ecosystem Features	Focus management on structural characteristics, organisms, or areas that represent important ‘underpinnings’ or ‘keystones’ of the current or future system of interest
E. Restore Structure & Function	Rebuild, modify or transform ecosystems that have been lost or compromised, in order to restore desired structures and functions
F. Protect Refugia	Protect areas less affected by climate change as sources of ‘seed’ for recovery or as destinations for climate-sensitive migrants
G. Relocate Organisms	Engage in human-facilitated transplanting of organisms from one location to another in order to bypass a barrier (e.g., conflicting current)

Within each general strategy, the Compendium provides a variety of ideas for management adaptation options for coral reefs, along with examples of climate-smart design considerations. In conjunction with available vulnerability information, these can provide a starting point for identifying any gaps in your current plan and crafting specific place-based actions to add to your list.

**Source:** ‘Adaptation Design Tool: Corals & Climate Adaptation Planning’, NOAA Technical Memorandum (Parker et al. 2017); www.coris.noaa.gov/activities/CCAP_design. Full Compendium available therein, and in West et al. (2016).) The example adaptation options and climate‐smart design considerations in the Compendium are meant to be illustrative rather than comprehensive and to stimulate ideas for site‐ releva nt po ss ibil itie s. As new re search a nd practice s e merge, th e rang e o f examples will continue to grow and the Compendium will need to be reviewed and updated over time.

### Box 2 Climate-Smart Design Considerations

For any management action to be considered Climate-Smart, two categories of Climate-Smart Design

Considerations must be applied:

**Category 1 Design Considerations**

How will climate change directly or indirectly affect how the stressor of concern impacts the system?

**Category 2 Design Considerations**

How will climate change affect the functionality of the management action (through effects on the stressor and/or effects on the action directly), and as a result how will the action need to be adjusted (in terms of location, timing, or engineering design)?

**Source:** ‘Adaptation Design Tool: Corals & Climate Adaptation Planning’, NOAA Technical Memorandum (Parker et al. 2017); www.coris.noaa.gov/activities/CCAP_design.

### Introduction to the Adaptation Design Tool

**Fig. 2:**
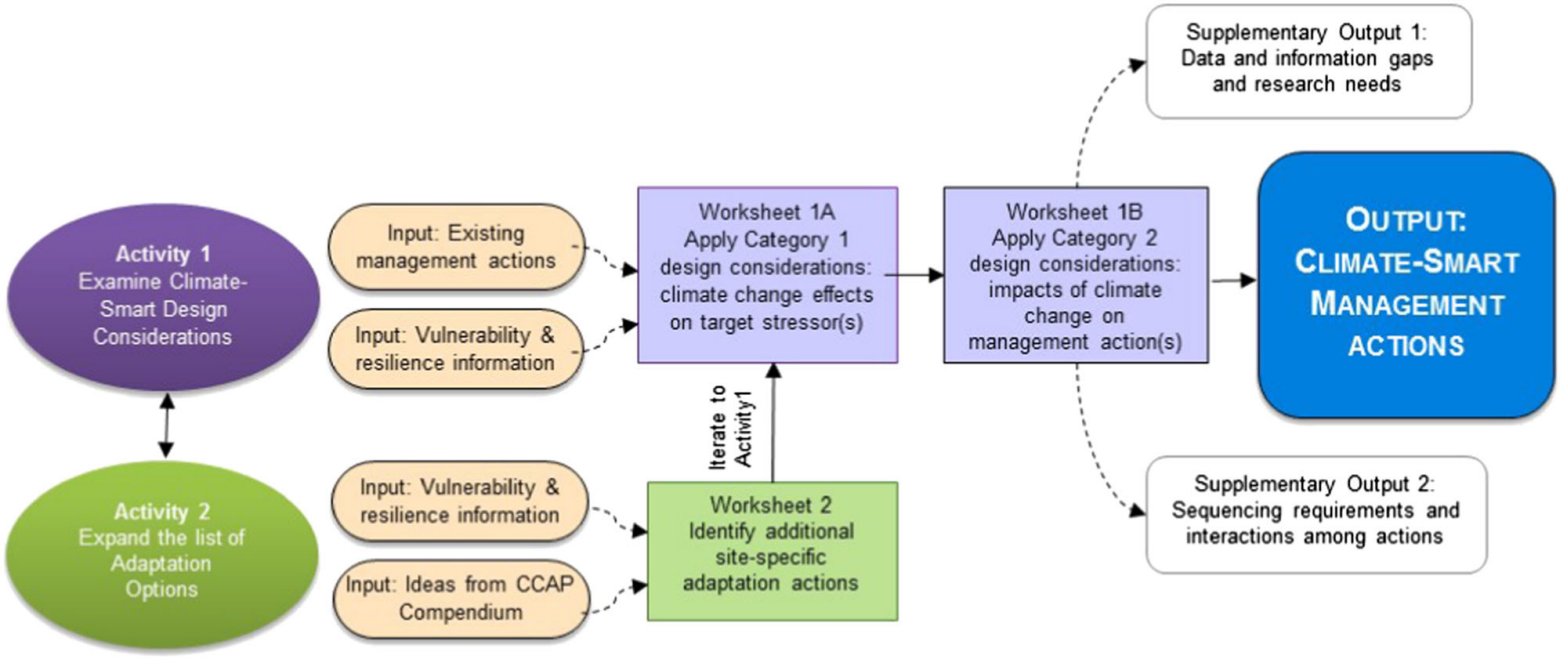
Flow Chart of Activities of the Adaptation Design Tool

The structure and flow of the Design Tool are shown in Fig. 2. For more detailed descriptions of tool protocols, please refer to the user guide (Parker et al. 2017). In brief, Fig. 2 depicts two activity work-streams that enable the user to apply information about climate vulnerabilities to the re-engineering or redesign of existing or planned management actions, and to the identification of new adaptation options.

#### Activity 1

Develop information to address climate-smart design considerations and apply it to adapt existing actions to account for climate change effects (see Table 2 Worksheet 1A and Table 3 Worksheet 1B).

#### Activity 2

Identify additional, general adaptation options that may be needed to more comprehensively address climate change impacts, tailor them as place-based actions, and add them as a second iteration of Activity 1 (see Table 4 Worksheet 2).

The ultimate output (Fig. 2) of the combined activities is a set of management actions that are described in terms of the design adjustments that will be necessary to ensure maximum effectiveness in the context of climate change. The subsequent process of evaluating and selecting (Step 5 of the planning cycle) from among the resulting set of climate-smart actions will determine which actions are ultimately selected for implementation. Furthermore, once a subset of priority actions has been selected, those actions can be run through Activity 1 a second time with specialists, to attain a more detailed level of design for actual implementation (Step 6).

The tool process can be initiated with either Activity 1 or Activity 2, depending on the manager’s preference. Activity 1 can be the starting point to make management activities in an existing plan more climate-smart through application of design considerations (Worksheets 1A and 1B). Alternatively, Activity 2 can be the starting point if the first priority is to identify actions (Worksheet 2) to fill gaps in the existing management plan due to unaddressed climate vulnerabilities, or if there is not yet an existing plan. In both cases, vulnerability and resilience information and the Compendium (West et al. 2016) are key inputs to the climate-smart design process. For information and resources on how to summarize existing vulnerability and resilience information, and to access the full Compendium of adaptation options, see the tool user guide (Parker et al. 2017).

What follows is a brief overview of the tool activities. Fully-completed examples are presented in the next section (‘Testing the Tool’, Tables 2–4). Please refer therein for the detailed column listings and heading definitions.

#### Activity 1

In this activity, two worksheets are used to apply climate-smart design considerations (Box 2) to existing management actions. Worksheet 1A (Table 2) guides coral reef managers through a series of information-gathering steps designed to address Category 1 design considerations about how climate change is expected to affect the stressor(s) that are being targeted by or accounted for in each management action. Worksheet 1B (Table 3) carries forward the outputs of Worksheet 1A to explore the implications of these stressor changes for the effectiveness of the management action, and consequently what adjustments are needed in location, timing, or structural design in order for it to remain effective given climate change.

The final output is captured at the end of Worksheet 1B as a redesigned version of the management action, which incorporates climate-smart design considerations. Also of key importance are insights that emerge during this thought process, which are recorded in the Notes columns of each worksheet. Documentation of reasoning, assumptions, information gaps, supporting science citations, and issues related to applying actions is of paramount importance, not only for immediate needs in design and implementation, but for future reference for adaptive management. Supplementary Output (SO) worksheets are offered to help organize this information so it can be carried forward to: inform data-gathering and research needs (SO1); and understand synergies, trade-offs and sequencing among actions (SO2), which is relevant for evaluation and selection of a final priority list. These SO worksheets are provided in the Design Tool user guide (Parker et al. 2017).

#### Activity 2

In this activity, Worksheet 2 (Table 4) is used to help identify actions that could be added to a management plan. Key vulnerabilities that are not sufficiently addressed in the existing plan can be examined, in conjunction with the Compendium, to brainstorm and craft actions to fill those gaps. Or, if there is no existing plan and it is being developed for the first time, Activity 2 is equally effective for brainstorming a list of actions from scratch. The CCAP Compendium developed for coral reefs (Box 1; Parker et al. (2017); West et al. (2016)) informs the activity by providing general adaptation strategies, adaptation options, and climate-smart design considerations compiled from an intensive review of the literature. The ecologically-based, general adaptation strategies provide a structure for considering the full range of available adaptation options. In Worksheet 2, general ideas are converted into specifically-defined actions appropriate to the particular reef’s unaddressed vulnerabilities. These are then run through Activity 1 in order to apply climate-smart design.

The culmination of these two activities--an expanded list of climate-smart actions—is intended to provide the fullest range of ideas possible for evaluation and selection (Step 5; Fig. 1) and for potential inclusion in a new or revised (more climate-smart) plan. The list is comprised of better-crafted actions with respect to climate change implications for timing, placement (siting), engineering, and other physical aspects of design. Sometimes, putting pre-existing actions through Activity 1 may reveal that some actions will no longer be feasible as climate changes. In that case, Activity 2 can be used in a more targeted manner to replace those eliminated actions.

In the next section, we present the results of expert consultations carried out with practitioners in Pacific and Caribbean reef locations. These results were used to improve the tool and draw lessons-learned for adaptation planning moving forward.

## Testing the Tool: Guánica Bay and West Maui Expert Consultations

### Background

Two consultations with experts in coral reef and watershed management were conducted to test the draft Design Tool. The goal of the expert consultations was to solicit feedback on the use and relevance of the tool in incorporating information on climate change vulnerability into coral reef management using specific case studies. These consultations were conducted in Guánica Bay, Puerto Rico (November 2015) and West Maui, Hawaiʻi (January 2016), areas identified as priority coral reef watersheds by the U.S. Coral Reef Task Force (USCRTF). These two settings (Table 1) represented a range of management contexts for testing the utility of the tool. Both consultations were 1 ½ days in length, allowing for several management actions to be put through the tool.

**Table 1:** Comparison of setting and planning context for testing the Adaptation Design Tool

Location Characteristics	Guánica Bay Watershed, Puerto Rico	West Maui, Hawaiʻi
Geographic setting	Single, highly modified watershed and adjacent bay and off-shore coral reefs	Multiple watersheds and adjacent exposed reef area
Key non-climate threats	• Upland erosion in the coffee growing regions • Reservoir sedimentation and transport • In-stream channel erosion • Loss of historic Guánica Lagoon • Legacy contaminants • Sewage treatment • Overexploitation of fishes^a^	• Upland erosion from former agricultural lands • Nutrient influx to coastal waters from waste-water injection wells • Runoff of urban district pollutants • Legacy ground water pollutants • Stream diversions • Illegal take of herbivores in the fishery restoration area • Unsustainable harvest outside the fishery restoration area
Planning framework	The Watershed Management Plan undergoing revision	Conservation Action Plan and three Watershed Management Plans undergoing expansion to two additional watersheds
Availability of information on vulnerability to climate change	Information on climate change and vulnerability summarized from multiple information sources including an island-wide vulnerability assessment for Puerto Rico (Puerto Rico Climate Change Council (PRCCC) 2013).	Information on climate change and vulnerability summarized from multiple information sources including the Pacific Islands Regional Climate Assessment (Keener et al. 2012).

^a^Stressor not addressed in Center for Watershed Protection (CWP) (2008); being addressed in the updated watershed management plan

The Guánica Bay watershed is highly modified, having been artificially increased in drainage area by a series of inter-watershed transfer channels, five reservoirs and two hydroelectric plants. It was designated as a priority watershed by the USCRTF because its associated coral reefs are threatened by extensive land-based sources of pollution. Therefore, coral reef protection and recovery efforts have a significant focus on watershed management. In 2008, the Center for Watershed Protection developed a watershed management plan (Center for Watershed Protection (CWP) 2008) outlining a comprehensive set of actions and an overall management strategy for improving and protecting the Guánica Bay watershed from pollution from land uses and alterations. In 2010–2012, the U.S. Environmental Protection Agency held a series of stakeholder workshops that resulted in a comprehensive list of potential management actions to supplement those in the 2008 plan that was undergoing revisions (Bradley et al. 2014; Bradley et al. 2016).

In West Maui, the planning scale consists of five watersheds and adjacent reefs. Management issues recognized by USCRTF priority watershed designation include nutrient inputs from injection wells, unsustainable fishing practices, and land-based pollutant runoff. A Conservation Action Plan for the reef area itself, plus three watershed management plans, have been developed through a multi-agency effort (Barger and Clark 2012; Hawaii Department of Land and Natural Resources et al. 2014; Sustainable Resources Group International 2012a; Sustainable Resources Group International 2012b). Planning for two additional watersheds was underway at the time of the consultation.

### Expert Consultation Preparation and Design

Prior to the consultations, available information on climate change vulnerability was assessed, and for both locations was found to be summarized in a manner that did not relate explicitly to the management actions being reviewed. Therefore, a summary vulnerability assessment table was created using the Local Early Action Planning (LEAP) Tool for climate change adaptation developed for the Coral Triangle region (U.S. Coral Triangle Initiative Support Program 2013). This was provided to each group as a resource (‘input’) when using the Design Tool.

In collaboration with the project team, each expert group selected 3–5 management actions from their existing plan, which they considered priorities for climate-smart design. The actions were selected because they had known sensitivities to climate change and had already been identified by the experts as priority actions for their plans. As such, the selected actions reflect the interest of the experts in building climate-smart knowledge in these areas and does not reflect opinions or advocacy on the part of the project team for use of these actions.

#### Guánica Consultation

A small group of watershed and coral reef experts participated in the consultation. The facilitator had not been involved in the development of the tool but had extensive knowledge of coral reef science and management in Puerto Rico, and therefore brought a fresh perspective to the test application. Prior to the consultation, the facilitator conducted several phone calls with the expert group to choose actions from the 2008 Watershed Management Plan and the EPA Workshops to run through the tool. There were two participants, a watershed manager and a coral reef manager. A member of the project team with a background in coral reef ecology and management also participated on the first day to ensure understanding of the tool. The consultation began with an overview of the Design Tool and the worksheets and an introduction to the CCAP Compendium. This overview was very important, but time-consuming. It was recommended that in future consultations, an overview could be provided as a webinar prior to the consultation. It was also suggested that a slightly larger group of experts would be optimal. These recommendations were adopted for the West Maui Consultation two months later.

#### West Maui consultation

Three webinars were held with reef and watershed managers to plan the consultation, identify participants and select actions for the tool. Webinars provided participants with an overview of the Design Tool and ensured that key inputs to the tool (vulnerability assessment and a list of management actions) were available and reviewed in advance of the consultation. The facilitator was a member of the project team with extensive knowledge of coral reef science and management in Hawai’i, as well as experience using the Design Tool. The management actions that were selected helped define the spatial and technical scope of the consultation, which in turn helped identify the experts needed (5 watershed managers and one coral reef scientist) for the in-person consultation.

For both consultations, the selected management actions were used to pre-populate columns 1 and 2 of worksheets 1A and 1B. Participants were asked to review the CCAP Compendium in advance of the consultation along with the draft Design Tool user guide. In particular, participants were asked to think about how existing management actions were aligned with the general adaptation strategies and to identify potential gaps.

## Results

The participants of both expert consultations successfully completed climate-smart designs for 3–5 actions using the Design Tool over the 1 ½ -day periods. The Guánica Bay and West Maui experts generated noteworthy findings for their respective management areas from Activities 1 and 2 and both supplemental worksheets. Example actions from each location are provided in Tables 2–4 and described in detail below, along with general feedback on using the worksheets.

**Table 2:** Worksheet 1A of the Adaptation Design Tool

Worksheet 1A apply category 1 design considerations: climate change effects on target stressor(s)							
	A1	A2	A3	A4	A5	A6	A7
	Action number	Existing management action	Target stressor(s)	Climate change effects on stressor(s): direction, magnitude, mechanism, uncertainty	Timing of climate change effects	Implications for effectiveness metrics and how to measure them	Notes
COLUMN DESCRIPTION	Provide a sequential ID number for each action.	List each site-specific action from your management plan and/or from Activity 2. (Color code actions from Activity 2 as ‘new’ actions being added to the original list of ‘existing’ actions.)	Identify the stressor(s) (e.g., pollutant, fishing pressure, etc.) that the management action targets. (An individual action may address more than one stressor.)	Describe expected climate change impacts on the target stressors. This includes information on the direction, magnitude, and mechanism of change along with level of uncertainty. This will support consideration of how actions would have to be modified (e.g., scaled, placed, timed, engineered) to remain effective. Supporting materials needed include vulnerability and resilience information, climate projections, etc.	Indicate the anticipated timing of when climate change will affect the target stressor(s). This informs when the action is needed, sequencing with other actions, and the time frame under which effectiveness should be evaluated. Mid-century is a management-relevant time frame commonly used; however, this also could include seasonal outlooks/forecasts, or shorter-term events like El Niño.	Identify metric(s) to be used to assess technical performance (i.e. effectiveness) of the action. If possible, suggest targets for quantitative or qualitative changes in the stressor/ metric that would be used to measure effectiveness. Describe how monitoring (e.g., frequency, location, duration, etc.) might need to be modified given climate change effects on the stressor.	Make notes on reasoning, concerns, or information gaps essential for: 1) Providing a transparent record of the thought process; 2) Keeping track of emerging insights into sequencing needs or interactions with other actions; 3) Identifying causal chains to social or ecological effects on the stressor that may cause feedback loops; 4) Recognizing the action's possible consequences for other human or ecological systems outside of the reef; 5) Describing uncertainties/knowledge gaps that need to be filled as new information becomes available.
ILLUSTRATIVE GUÁNICA BAY RESULT	1	Establish coral nurseries	• Land-based stressors (nutrients, sediments, etc.) • Sea surface temperature (SST) increases • Ocean acidification	• Land-based stressors will increase with increased rainfall. Magnitude unknown. • SST projected to exceed bleaching threshold for over half the year • New stressor – ocean/coastal acidification	• Precipitation changes (and ensuing consequences, such as erosion) occurring now • Larger storms have already become more frequent	• Need to monitor success of the basins during larger storms • Effectiveness metrics: number of corals in nurseries, time corals spend in nurseries until outplanting • Need to scale up the number of nurseries to balance increasing loss of corals • Coral residence time in nurseries should be shortened to produce more corals for outplanting • Coral survival in nurseries may be more episodic—large-scale death from bleaching or storms. Thus, monitoring must occur after adverse events.	• Nurseries may sequester carbon; additional research needed • Changes in ocean acidity will be spatially highly variable but changes in temperature will be relatively more uniform • Need to do a better job with land-based sources of pollution to compensate for SST stress
ILLUSTRATIVE WEST MAUI RESULT	2	Establish stormwater infiltration basins in urban areas to remove contaminants from stormwater and recharge ground water	• Debris, sediment, nutrients, other contaminants (e.g. pesticides, herbicides) adsorbed on the soil	• Precipitation depends on the model: decreased rainfall during the wet season (5–20% (statistical models)); or, increased rainfall during the wet season (0–25%) and wetter in dry season (dynamical models) • Location in watersheds also affects precipitation projections. Overall, high uncertainty about precipitation. • Change in stream flow caused by change in precipitation • Increased frequency of storm events	• SST consequences have already occurred but will intensify in the future • Ocean acidification not a major problem yet but could be in coming decades	• Effectiveness metric is what percent of water infiltrates to ground water and what percent of contaminants is removed • Smaller percent of water may infiltrate because water may overtop the basin	• Infiltration basins near the coast will be less effective under sea level rise • Larger standing pools in infiltration basins could increase mosquito abundances • Need greater consensus among precipitation models • Increased water and energy demands under climate change may produce additional contaminants

**Table 3:** Worksheet 1B of the Adaptation Design Tool

Worksheet 1B apply category 2 design considerations: impacts of climate change on management actions								
	B1	B2	B3	B4	B5	B6	B7	B8
	Action number	Existing management action	Changes in effectiveness of management action due to: climate impacts on target stressor	Changes in effectiveness of management action due to: climate impacts on management action	Time frame or constraint for using the action and implementation (e.g., urgency, longer or shorter term)	What changes are needed to adapt the action (place, time, and engineering design)	Climate-Smart Management Action	Notes
COLUMN DESCRIPTION	Transfer action numbers from Worksheet 1A.	Transfer management actions from Worksheet 1A (including any management actions identified because of Activity 2).	Describe how climate impacts on the stressor will change the effectiveness of the management action over its implementation and functional lifetime. Will the action be able to handle changes in the target stressor?	For actions that involve physical structures or elements, describe how climate change may directly impact the management action in ways that will change the effectiveness of the management action over its implementation and functional lifetime. Could the action be physically destroyed by climate change impacts?	Identify temporal considerations, including: (1) urgency due to anticipated time frame of climate change effects on the action and (2) short-term and long-term needs for planning and implementation of the action (including lead-time for design, permitting, construction, or other enabling conditions).	Describe the changes needed to adapt the design of the action in terms of place, time and engineering design. Be sure to review and consider the information from all previous columns including the Notes columns.	Revise the original management action (from Column B2) to incorporate the climate-smart design considerations. Be as specific as possible.	Make notes to: 1) Provide a transparent record of the thought process; 2) Identify knowledge gaps and research needs for better understanding climate impacts and design needs/changes in effectiveness of the action; 3) Record social or economic considerations for making adaptation design changes to the action; 4) Describe any other concerns or uncertainties relevant to adaptation design of the action.
ILLUSTRATIVE GUÁNICA BAY RESULT	1	Establish coral nurseries	• Survival of nursery corals may be reduced due to increased smothering by sediment • Increased SST could bleach and kill nursery corals • Ocean acidification may reduce growth of nursery corals	• Entire nurseries could be destroyed by large storms • Access to nurseries could be limited by an increase in stormy days	• Urgent – huge loss of reefs	• Choose strains/clades that are resilient to high temperatures, ocean acidification, and land-based pollutants • Pipe to bring cooler water up to nursery area • Should add additional coral species, including *Porites*, *Montastraea cavernosa*, and *Orbicella spp*. • Design that allows nursery to be detached and moved in cases of storm events	Establish coral nurseries with corals resilient to high temperatures, ocean acidification, and land-based pollutants. Use a wide range of species. Pipe cooler water from greater depths to nursery to prevent heat stress during potential bleaching periods. Nurseries should be movable to protect them from the increasingly frequent, large storms that are expected.	• Additional long-term benefit of shoreline protection if reefs were restored • Floating structures that do not affect existing habitats? • Need to know the minimal size of fragments to be successful • Research for intervention actions for nurseries during bleaching events • Also consider not releasing sewage or stormwater during elevated SST
ILLUSTRATIVE WEST MAUI RESULT	2	Establish stormwater infiltration basins in urban areas to remove contaminants from stormwater and recharge ground water	• If higher flows, basins may not be able to contain stormwater • Effectiveness depends on changes in sediment load • Fine sediments could clog infiltration basins • If lower flows, current basins will be larger than necessary	• Basins may be washed away in larger storms • If too much rain, basins might become ponds • Less effective with SLR if water table changes	• Urgent – to stop impacts of urban pollutants on the reef • Could only be considered for new developments and developments early in design	• Adjust outflow pipe to handle larger precipitation events • Put drains with lids on the bottom to quickly empty basins if flooding is imminent • Don’t establish in SLR inundation areas or too near the water table • Incorporate early in planning process	Establish infiltration basins designed to accommodate larger runoff volumes and sediment loads, unless smaller runoff volumes seem more likely. Clean accumulated sediment from basins more often to prevent clogging and be prepared to reconstruct basins or parts of basins after larger storms. Basins should have draining mechanism to keep from overflowing and inundating surrounding area. Basins should be created outside the area likely to be affected by SLR. Increased ponding of detained stormwater due to larger storms should be prevented in order to prevent them from becoming mosquito breeding grounds.	• Will climate change affect ground water, and how would those changes interact with infiltration basins? • Mosquito borne diseases may increase— an unintended consequence

**Table 4:** Worksheet 2 of the Adaptation Design Tool

Worksheet 2 Identify additional site-specific adaptation actions				
COLUMN DESCRIPTION	1	2	3	4
	General Adaptation Strategy	Definition	Potential New Site-Specific Action	Key Vulnerabilities Addressed
	General Adaptation Strategies from the CCAP Compendium	Definition of general strategies, placed in the context of future as well as current conditions, including climate-related impacts.	Craft new, potential place-based actions (i.e., general options from the Compendium, made into actions specific to your location) to address gaps in existing/planned activities. Transfer these actions to column 2 of Worksheet 1A as additions to run them through Activity 1; color code these actions to distinguish them as ‘new’ actions being added to the original list of ‘existing’ actions.	Describe what key vulnerabilities are being addressed by this action (that may not have been sufficiently addressed in your plan thus far). This documents the logic for why the action is needed/how it addresses the impacts of particular climate stressor-interactions as identified in the vulnerability information.
ILLUSTRATIVE GUÁNICA BAY RESULT	A. Reduce non-climate stresses	Minimize localized human stressors (e.g., pollution, fishing pressure, coastal development) that hinder the ability of species or ecosystems to withstand or adjust to climatic events	• Convert all homes with on-site sewage disposal systems to tertiary level waste-water treatment • Convert all waste-water treatment plants to tertiary treatment levels	Sea level, rainfall, extremes, hurricanes and storms
ILLUSTRATIVE WEST MAUI RESULT	B. Protect key ecosystem features	Focus management on structural characteristics (e.g., geophysical stage), organisms, or areas (e.g., spawning sites) that represent important ‘underpinnings’ or ‘keystones’ of the current or future system of interest)	• Expand or duplicate the herbivore replenishment areas in reefs in the 5 watersheds and adjacent source areas in Olowalu, North Kihei • Protect some of the worst reef areas (reefs that have survived multiple stressors) as being resilient to multiple stressors	Coral bleaching events that impacted reefs in 2014–2015

### Activity 1

#### Worksheet 1A: application and feedback

Using Worksheet 1A (Table 2), participants examined the impacts of climate change on stressors addressed by or accounted for in the selected management actions. They were able to use the prepared vulnerability assessments to document the implications of climate change for those target stressors.

For Guánica Bay, the 2010 Guánica Decision Workshop yielded many ideas for management actions in addition to those in the 2008 Guánica Bay Watershed Management Plan. One of these–establish coral nurseries–was chosen for the consultation. There has already been significant loss of habitat for mobile species as a result of land-based stressors (nutrients, sediments, etc.) and increasing sea surface temperature anomalies. The participants identified that climate change will likely cause increased intensity of rainfall events, exacerbating the land-based stressors. Also, elevated sea surface temperatures exceeding the bleaching threshold may soon occur during half of each year. This information was documented in Worksheet 1A.

In the case of West Maui, there was a plan to establish infiltration basins in low-lying urban areas to limit sediments, nutrients, and other contaminants from entering near-shore waters. This was because increased precipitation and incidences of severe storm-runoff due to climate change were deemed likely to result in increased sedimentation and nutrient runoff above and beyond what had been historically seen. Different models, however, predicted vastly different future levels of precipitation—some wetter, others drier. These different projections were documented in Worksheet 1A. Participants highlighted the importance of capturing such differing scenarios and carrying them through the climate-smart design process. Documenting alternative future conditions explicitly accounts for location-specific uncertainties, and supports a flexible and adaptive approach.

#### Worksheet 1B: application and feedback

Using Worksheet 1B (Table 3), participants carried forward their analyses from Worksheet 1A to evaluate the implications for the design of effective management actions in the face of climate change impacts. This culminated in a restatement of the existing or planned management action that incorporated everything from both worksheets that should be included in climate-smart design.

All participants agreed that the information developed in Worksheet 1B was extremely valuable. They found it especially useful to be provided a series of smaller steps to achieve a final synthetic restatement of the management action incorporating consideration of climate change. They recognized that this can lead to important new insights about effective use of management actions. The Guánica Bay group identified the need to choose coral strains/clades that are temperature resilient and to add more reef-building coral species (in addition to Acroporids) for their coral nursery action. For West Maui, after incorporating climate-smart design considerations, the expert team identified the need to not only re-engineer the basin design for future runoff, but also to incorporate a new design element to address an anticipated climate change effect--drainage of the basin to mitigate potential disease vectors, such as mosquitoes, that could emerge as a result of ground water inundation of low lying coastal areas due to increasing air temperatures and rising sea levels.

### Supplemental Output Worksheets Application and Feedback

In discussions of Supplementary Output 1 (information and data gaps), Guánica Bay participants noted a need to research ways to build coral nurseries that could be moved around during storm events to avoid their destruction. They also recommended assessment of how several land-based coral nurseries could be established in Puerto Rico to address the Territory-wide scale of coral loss. Participants of the West Maui consultation identified the need for observational research and modeling of projected rainfall (spatial and temporal patterns as well as intensities) as critical for improving climate-smart adaptation actions. This is an example of how considering climate change influences on the stressors and the actions caused participants to recognize information gaps that would prompt a return to the vulnerability assessment step (Fig. 1; Step 2) of the climate-smart cycle.

Regarding Supplementary Output 2 (interactions among actions), participants affirmed the usefulness of examining synergies, trade-offs and sequencing among management actions. For Guánica Bay, establishing coral nurseries was deemed synergistic with capture and nursery-raising of larval fish of target species to replenish depleted populations, including herbivores that help maintain favorable habitat through grazing. Both actions were also considered critically dependent on establishment of protected areas to ensure effective protection and management of reefs to which these coral and fish populations are restored. For West Maui, interactions among several adaptation options were recognized as important to support climate-smart adaptation. The success of infiltration basins will depend on simultaneous actions to reduce sediment load by stabilizing streambanks and reforesting fallow agricultural lands. In terms of sequencing, changes in county government rules would be needed first to require on-site stormwater infiltration for all new construction.

### Activity 2

#### Worksheet 2: application and feedback

Using Worksheet 2 (Table 4), participants identified new adaptation options that filled gaps in plan coverage by identifying vulnerabilities not addressed by current management actions. Using the CCAP Compendium (Parker et al. 2017; West et al. 2016) along with the summary vulnerability assessments provided, participants converted general adaptation options into place-based actions appropriate for their management context.

The Guánica Bay participants reviewed the Compendium and identified additional adaptation options that would contribute to more comprehensively addressing climate change impacts, and tailored them as place-based actions specific to the Guánica Bay Watershed. For example, the group felt that sewage and waste water treatment problems are major concerns in coastal and island communities. This stimulated the identification of an action to ‘convert all homes with on-site sewage disposal systems to tertiary level waste-water treatment’.

The West Maui participants recognized that the current plan did not include actions to address the vulnerability of coral reefs to bleaching events, such as that which occurred during the 2014–2015 El Niño. As a result, the group identified new management actions, which included expanding or duplicating herbivore replenishment areas in reefs in the five watersheds and adjacent source areas and targeting for protection those reefs with a history of exposures to multiple stressors such that they may have acclimated or adapted to challenging conditions.

### Insights and Recommendations for Using the Tool

Overall, the process structured by the Design Tool was well-received by the participants of both expert consultations. No overhaul of the process or worksheets was recommended. However, after both consultations had been completed, a comparison of general comments and recommendations led to some refinements of the Design Tool and development of a user guide (Parker et al. 2017). This included key insights and recommendations that fall into the following three general themes: (1) improving effectiveness and efficiency in using the tool, (2) valuing the process as being equally important as the ultimate outputs, and (3) addressing overarching considerations such as matching temporal and spatial scales, identifying and documenting uncertainty, and integrating outputs into other planning and decision-making processes. Each of these themes is discussed below.

### Improving Effectiveness and Efficiency in Using the Tool

The Design Tool can be effective at the following two levels of application for climate-smart design: (1) rapid and (2) in-depth. Overall efficiency is maximized by selecting the best type of effort for your need. Under the rapid (or ‘rough cut’) application, an individual manager or small group can put a broad range of actions through the tool quickly, for a basic understanding of the functionality and adaptability of each action under climate change; the goal is to develop sufficient information to support credible evaluation and selection under Step 5, or as a precursor to a more in-depth assessment. The in-depth application consists of a more comprehensive group effort, in which one or more expert panels would delve into greater detail for a subset of actions, in order to support more rigorous analysis of design needs; the goal in this case is to develop detailed specifications for implementation.

The process of reviewing many management actions using the tool can be a significant time investment. To increase efficiency, the following two recommendations were proposed during the consultations: (1) screening management actions to identify those that should take precedence for tool application, and (2) grouping management actions that target the same stressor(s) and sources. Actions can be screened for precedence if they and the stressors they address will be significantly affected by climate change; actions that are not much affected by climate change will not benefit from going through the tool (e.g., education campaigns for keeping beaches clean). Then the actions can be further screened for their perceived importance (e.g., by stakeholders or lawmakers) or urgency (e.g., due to funding availability or post-extreme event opportunity), or other criteria; thus, certain actions would be put through the tool first, representing an initial batch of climate-smart actions. Such screening must be balanced by retaining actions that may not appear to be priority actions but might become so if climate change considerations were incorporated into them. Ideally, all possible actions should have climate smart-design considerations applied (Step 4 of the climate-smart cycle) and be part of a structured evaluation and selection process (Step 5). However, the concept of screening management actions can be important under time constraints; this was emphasized in the West Maui consultation, reflecting the participants’ experience of having to incorporate a very large number of recommended actions into an integrated watershed-to-coral-reef management plan.

The other efficiency concept, also raised in the West Maui consultation, was grouping actions that address similar stressors (e.g., excess sediment runoff) and sources (e.g., erosion from agricultural fields), which would be similarly affected by climate change. This can minimize potentially redundant efforts in filling out worksheet columns that will have similar answers. This must be balanced with caution against consolidating actions that will be too dissimilarly affected by climate change, which would lead to overly generalized responses to the adaptation design considerations and impair the process of developing climate-smart redesigns.

### Valuing the Process of Using the Tool

Undertaking the full process of using the tool provides value to planning beyond just the climate-smart redesign of individual actions. The tool process highlights gaps in information or in planned actions that might otherwise have been missed. Including citations in the spreadsheets can help users compile a list of relevant resources and show where foundational science is lacking or ample. And while each action is reviewed separately, the process of scrutinizing action-related stressor, climate, and other environmental information often yields insights on potential interactions with other actions (e.g., synergies, trade-offs, linked interdependencies), as well as sequencing or timing needs. This information, which is captured in the ‘Notes’ columns of the worksheets, can be of great value in future formulation of an implementation plan. It advances a systems-based understanding by crossing the boundaries between actions to convey the idea that actions are not performed in a vacuum and are part of a larger system. The value of this aspect was apparent in Guánica Bay, where management planning and plan revision were sometimes isolated from close interactions with technical experts (e.g., climate scientists, watershed scientists) who could contribute inter-disciplinary expertise to the review and revision process. Having experts with knowledge across a range of inter-related system components results in learning about other parts of the system in ways that generate new insights.

The tool establishes an iterative process at multiple levels, between activities within the tool and among steps throughout the climate-smart planning cycle. When using each worksheet within an activity, the thinking required to fill out later columns can generate insights that cause users to return to earlier columns, or even to other actions. Likewise, working on later worksheets may cause users to return to an earlier worksheet to add more information there. Therefore, while a linear progression through the tool will generate an ‘answer’ (an action revised to be climate-smart), it is the opportunity afforded by the iterative process to focus and refine management-relevant information that is beneficial, regardless of initial entry point.

Use of the tool also highlights information needs and produces insights that prompt a return to earlier or later steps in the climate-smart planning cycle, which might not otherwise be revisited. For example, a common experience while testing the tool was that the scale and/or level of detail of vulnerability information typically available from Step 2 (Vulnerability Assessment) was not sufficient to address the questions that the tool poses about climate change effects on the site-specific actions and associated stressors. Common deficiencies were that the existing summary of climate vulnerability information was too general, large-scale, long-term, non-specific in terms of time frame, and/or non-specific in terms of spatial distribution to be relevant to the action being considered. Thus, the process of using the tool helped inform the specific needs for revised climate vulnerability information and thus directed attention back to Step 2 while maintaining progress on Step 4.

Progressive application of the tool can help generate a better grasp of what can (and cannot) be achieved through a set of management actions in the face of climate change. Given the importance of setting realistic, achievable goals and objectives, the process of using the tool may prompt a return to Step 3 to revise management objectives. Use of the tool often generates ‘ancillary’ information that is relevant to future planning steps, including evaluation and selection (Step 5), or plan formulation and implementation (Step 6), as well as directly elicited information on performance targets and metrics that are moved forward to Step 7 (Monitoring). For example, information is often captured that relates to criteria typically used in evaluation and selection, such as economic feasibility, flexibility, urgency, or social or political barriers. Similarly, as has been mentioned, the tool process captures information on project interactions, sequencing and timing that is brought forward to Step 6. Furthermore, the tool itself can be used to develop more in-depth design information for Step 6.

Climate-smart strategies and design principles focus on climate; however, other global changes such as population growth and development trajectories often have important interactions and feedbacks with climate change effects, as well as critical influences on what adaptation methods and locations are feasible and effective. The tool lends itself to inclusion of these other drivers of global change, which are often presented in vulnerability assessments. A key outcome of the tool process is to refocus resource management planning and incorporate consideration of future conditions, including other important global changes.

### Overarching Considerations in Using the Tool

In developing, testing, and refining the Design Tool, overarching considerations were identified regarding the following: (1) dealing with uncertainty, (2) matching temporal and spatial scales, and (3) integrating the outputs of the tool into other planning and decision-making processes. For the first consideration, uncertainties in both magnitude and direction of a change can affect the vulnerability assessment, how actions are adapted, and ultimately what adaptation strategies are selected. In some cases, divergent climate change projections for the area of interest (e.g., the area will get wetter under some scenarios but drier under others, as in the West Maui) must be considered. Uncertainty surrounding climate change projections must therefore be incorporated into the Design Tool in Activity 1, and there are multiple options for accomplishing this. For the case of diverging climate projections, actions that will be affected by this uncertainty can be given multiple rows in the tool worksheets—one for each alternative scenario. For such divergent scenarios, each scenario can be expected to require unique responses to the climate-smart design questions, and thus can be addressed separately. Alternatively, if there are only two highly divergent scenarios, they can be addressed in a single row of the tool with information for each scenario included in that one row. Or, if one scenario has more serious implications than another, users may choose to focus first on the scenario with the greatest ‘downside’. In all cases, uncertainties should be documented and characterized to the extent possible in the worksheets. For cases where climate projections may not diverge in direction but have high uncertainties in the magnitude of the projected change, the nature of the uncertainty must be carried through the design considerations to arrive at what revisions to the action (e.g., redesign or relocation) would be needed for the action to continue to function within the bounds of that uncertainty. To the extent that a high level of uncertainty may not be able to be fully accounted for through redesign, the implications of the uncertainty should be noted for consideration in Step 5 (evaluation and selection) of the planning cycle, potentially through a risk assessment.

The second consideration is matching the scale of climate change information to the temporal and spatial scale of the actions going through the tool. Outputs of vulnerability information from Step 2 of the planning cycle must specifically inform the tool worksheets. There are many options for how to conduct and summarize outputs from a climate change vulnerability assessment, and a particular approach is not required for using this tool. However, the match in scale of information between the management action and the relevant climate change effects is critical. Both the West Maui and Guánica experts noted this was important. In practice, there are often difficulties with obtaining appropriately scaled climate information. For instance, climate change models downscaled to the resolution at which management decisions are occurring may not be available, and similarly, there may not be climate projections at management-relevant time scales. Moreover, it could be discovered that in light of anticipated climate changes, some actions may need to be implemented at larger scales than managers have been considering previously (for example, increasing spatial scale to capture pathways of intra- or inter-system connectivity). Vulnerability information would need to be matched to that larger scale, as well. Nevertheless, whatever location-specific and time-relevant climate vulnerability information is available should be considered. It is preferable to proceed with whatever information is available rather than wait for ideal or complete information.

The third consideration is the ability to use the tool in concert with different planning processes, and for different ecosystems. It supports structured decision-making (SDM) by being inclusive, transparent, and systematic. It promotes inclusive planning by convening stakeholders from different areas of expertise, and giving each an equal voice. While there are places and times when small groups of experts can use the tool, given the importance of inclusivity to the ultimate acceptance and success of a decision process, there are also places and times when a broader group of stakeholders would be beneficial. The tool promotes transparency because as an explicit component of the process, users document their decisions and associated supporting information in a consistent manner. This is valuable not only because it provides a mechanism for other managers and future decision makers to understand and replicate the process, but also because it helps establish a common understanding and acceptance of the associated decisions, thus building support as the process moves forward toward implementation. It is systematic because it standardizes the adaptation thought process and records the logic used to identify adaptation options. Collectively, this facilitates revisiting previous adaptation decisions in light of new information and technology, supports organizational continuity, and increases the legitimacy of the decisions.

Finally, the tool can be used for non-coral reef systems with little modification. The concepts behind the column headings in the tables are applicable to other systems, such as watersheds, wetlands, or estuaries, although the terminology used may need minor adjustments. The major alteration needed for application to non-coral systems would be the CCAP Compendium. This was developed specifically for coral reefs, though managers from a non-coral system could apply the ideas of the Compendium in an analogous way to their own systems. Alternatively, they could do their own literature review to make their own system-specific Compendium, or gather ideas directly from experts via expert elicitation. The transferability of the tool is already being recognized by other organizations who have recently taken it up for use in management planning for wetland habitat, submerged aquatic vegetation and toxic contaminants.

## Conclusions

The overall intent of the Adaptation Design Tool is to catalyze transformational thinking on how to convert climate change science to adaptation action on the ground. In the past, a business-as-usual approach to adaptation planning has often overlooked the need to redesign or add new actions based on key vulnerabilities and has thus resulted in a limited range of adaptation options. Through incremental steps, the tool helps users collate and document the knowns and unknowns in order to generate an expanded portfolio of climate-smart ideas—each explicitly crafted for most effective timing, location, and structural design--to support a more rigorous evaluation and selection of priority actions. By laying out a logical, step-wise process for linking vulnerability-to-impacts-to-adaptation, the tool helps scientists, natural resource managers, and other stakeholders work together to apply the best available science to brainstorm and design effective actions. This ‘logic chain’ concept is consistent with other assessment processes employed by EPA and other resource management agencies. Examples include causal assessment and the EPA Causal Analysis/Diagnosis Decision Information System tool (Barbour et al. 2004; Norton et al. 2014; https://www.epa.gov/caddis); and risk assessment (Kunreuther et al. 2013; National Research Council (NRC) 2009). However, these are retrospective in that they are diagnostic of past causes of current conditions. In contrast, the Design Tool is forward-looking, addressing the challenge of future climate change impacts that are otherwise difficult to tackle in a systematic way. This approach makes an overwhelming process more tractable through small steps and documents the best available information and solutions, while also revealing where information needs to be further developed for continued improvement of our actions. These small steps also encourage users to follow the logic chain in its entirety and not skip steps in the progression based on assumptions that could lead to partial or incorrect results. Overall, this transparent process facilitates better decisions now and in the future and provides a record that can be revisited when conditions change.

Because the Design Tool’s framework supports, strengthens and informs all steps in the climate-smart planning cycle, including revision of goals and objectives, it can also inform higher, strategic levels of planning. Some organizations, such as the Chesapeake Bay Program, are using the tool to consider adjustments to their strategic (The Chesapeake Bay Trust, 2018). In general, a strategic plan comes into being in the first place because of a recognized need to manage threats to a natural resource from existing stressors, in order to meet goals and objectives for maintaining the resource; and presumably, there are management actions that have been identified to address those stressors. Therefore, the way to determine whether adjustments will be needed to higher-level strategies is to first assess whether the current actions (and any new ones that might be added) will or will not be able to be made climate-smart such that management objectives can be met under climate change. If the answer to this question is ‘no’, then changes in objectives, goals, and the strategies themselves may be warranted. Overall, this underscores the fluid nature of adaptation planning as an iterative and often nonlinear process that is not a ‘one-off’ exercise. This is the ‘new normal’ of management planning, where a structured approach is used to document collaborative knowledge sharing and uncertainty as a foundation for adaptive management through time.

The challenge that the Design Tool tackles—supporting conversion of science to action on the ground--is essential but difficult. Research on the topic of producing useful science suggests that information should be developed through inclusive processes (for legitimacy), applied specifically to the decision makers’ questions (for saliency), and documented thoroughly and transparently (for credibility) (Cash et al. 2002; Hegger et al. 2012). Employing these attributes of information production is particularly important when addressing adaptation planning and design. Large barriers to action are created by uncertainties surrounding climate change projections, coarse spatial and temporal scales at which they are produced, and difficulty translating climate projections into impacts that managers can use to design adaptation responses. The Design Tool addresses these difficulties by facilitating a dialogue among scientists, coral reef managers, and other stakeholders to improve existing and identify new management strategies to address climate change realities. This dialogue helps identify deficiencies in existing climate vulnerability information and supports an iterative process of assessment, review, and refinement. Rather than ignoring climate change uncertainties, participants are encouraged to explore their effect on adaptation selection and design, and document changes in potential responses to the climate-smart design questions. The tool provides a platform for diverse views and opinions to be expressed, explored and recorded. Participants engage in learning through iterative dialogue, leading to successful joint knowledge production that is incorporated into adaptation planning and design (Hegger et al. 2012). The documentation of these thought processes is a defining feature of the tool. Through use of this tool, adaptation implementation is supported by providing salient, legitimate and credible information that accounts for uncertainties in climate change projections and impacts.

## Disclaimer

The views expressed in this article are those of the authors and do not necessarily reflect views or policies of the U.S. Environmental Protection Agency or National Oceanic and Atmospheric Administration.

## Footnotes

1 Please see West et al. (2016) for additional key background on the conceptual foundations of this work, and Parker et al. (2017) user guide for more detailed step-by-step instructions on tool application.
